# Supplementary material for: Contrasting Evolutionary Dynamics and Global Dissemination of the DNA-A and DNA-B Components of Watermelon Chlorotic Stunt Virus
Source: Viruses. 2025 Nov 30;17(12):1571. doi: 10.3390/v17121571 (PMC12737365; doi:10.3390/v17121571)
Supplement: Supplementary file 1 [file viruses-17-01571-s001.zip › viruses-3916436-supplementary.pdf]

Supplementary data

Contrasting Evolutionary Dynamics and Global Dissemination of the DNA-A and DNA-B Components of Watermelon chlorotic stunt virus

Zafar Iqbal

Central Laboratories, King Faisal University, Al-Ahsa P.O. Box 31982, Saudi Arabia; zafar@kfu.edu.sa; Tel.: +966-580-776-536

Table S1. Accession numbers, year of isolation/report, host plant, and country of isolation of WmA and WmB used in the study

No.	WmA	WmB
1	JX131283:WmA:2011:WildMustard:Jordan	AJ012082:WmB:1998:Watermelon:UK
2	KJ939448:WmA:2014:Leith:Watermelon:SaudiArabia	AJ245651:WmB:1999:Watermelon:Sudan
3	KC462552:WmA:2010:Watermelon:Palestine	AJ245653:WmB:1999:Watermelon:Iran
4	PQ399661:WmA:2023:Arizona:Melon:USA	EF201810:WmB:2006:Watermelon:Isreal
5	PP320241:WmA:2017:AlAhsa:Cucumber:SaudiArabia	EU561236:WmB:2008:Watermelon:Jordan
6	PP320240:WmA:2017:AlAhsah:Cucumber:SaudiArabia	HE800539:WmB:2012:Cucurbit:Oman
7	MK649819:WmA:2016:Muscat:Cucumber:Oman	HG941655:WmB:2013:Squash:Oman
8	MK649818:WmA:2016:Muscat:Cucumber:Oman	HG969288:WmB:2013:Watermelon:Oman
9	MH329672:WmA:2016:Muscat:Cucurbit:Oman	HM368372:WmB:2009:Melon:Jordan
10	MH329671:WmA:2016:Muscat:Cucumber:Oman	JX131284:WmB:2011:WildMustard:Jordan
11	MH329670:WmA:2016:Muscat:Cucumber:Oman	KC462553:WmB:2010:Watermelon:Palestine
12	NC 003708:WmA:1998:Watermelon:USA	KJ939447:WmB:2012:Watermelon:leith:Saudi Arabia
13	KY124280:WmA:2012:Watermelon:Mexico	KT272766:WmB:2010:Melon:Iran
14	KY488568:WmA:2016:Datura:Sudan	KT272768:WmB:2010:Melon:Iran
15	KT272771:WmA:2010:Watermelon:Iran	KT272770:WmB:2010:Melon:Iran
16	KT272769:WmA:2010:Watermelon:Iran	KT272772:WmB:2010:Melon:Iran
17	KT272767:WmA:2010:Watermelon:Iran	KU360594:WmB:2012:Zucchini:Saudi Arabia
18	KT272765:WmA:2010:Watermelon:Iran	KU360595:WmB:2012:Zucchini:Saudi Arabia
19	KM066100:WmA:2014:Watermelon:Leith:SaudiArabia	KY124281:WmB:2012:Watermelon:Mexico
20	KJ854919:WmA:2010:Watermelon:Palestine	KY488569:WmB:2016:Datura:Sudan
21	KJ854918:WmA:2010:Watermelon:Palestine	KY825716:WmB:2015:Brapa:Iran
22	KJ854917:WmA:2010:Watermelon:Palestine	MH329673:WmB:2016:Cucumber:Oman
23	KJ854916:WmA:2010:Watermelon:Palestine	MH329674:WmB:2016:Cucumber:Oman
24	KJ854915:WmA:2010:Watermelon:Palestine	MH329675:WmB:2016:Cucumber:Oman
25	KJ854914:WmA:2010:Watermelon:Palestine	MK649820:WmB:2016:Cucumber:Oman
26	KJ854913:WmA:2010:Watermelon:Palestine	MK649821:WmB:2016:Cucumber:Oman
27	KJ854912:WmA:2010:Watermelon:Palestine	MW588415:WmB:2006:Consolea spinosissima:USA
28	KJ854911:WmA:2010:Watermelon:Palestine	MW588416:WmB:2006:Optunia auberi:Mexico
29	HM368371:WmA:2009:Melon:Lebanon	MW588417:WmB:2006:Cactus:USA
30	EU561237:WmA:2008:Watermelon:Jordan	NC 003709:WmB:1998:Watermelon:UK
31	EF201809:WmA:2006:Watermelon:Israel	OK058530:WmB:2021:Watermelon:Germany
32	PP622787:WmA:2021:Watermelon:Mexico	OL416208:WmB:2019:Cucumber:Saudi Arabia
33	PP622786:WmA:2021:Watermelon:Mexico	OR865138:WmB:2019:Cucumber:Saudi Arabia

34	PP622784:WmA:2021:Melon:Mexico	OR865140:WmB:2019:Cucumber:Saudi Arabia
35	OR865133:WmA:2019:Cucumber:AlAhsa:SaudiArabia	OR865141:WmB:2019:Cucumber:Saudi Arabia
36	OR865132:WmA:2019:Cucumber:AlAhsa:SaudiArabia	PP320242:WmB:2017:Cucumber:Saudi Arabia
37	OR865131:WmA:2019:Cucumber:AlAhsa:SaudiArabia	PP320243:WmB:2017:Cucumber:Saudi Arabia
38	OR865130:WmA:2019:Cucumber:AlAhsa:SaudiArabia	PP622788:WmB:2021:Melon:Mexico
39	OR865129:WmA:2019:Cucumber:AlAhsa:SaudiArabia	PP622789:WmB:2021:Melon:Mexico
40	OL416207:WmA:2019:Cucumber:AlAhsa:SaudiArabia	PP622790:WmB:2021:Melon:Mexico
41	KY825715:WmA:2015:B.rapa:Iran	PP622791:WmB:2021:Watermelon:Mexico
42	HG969278:WmA:2013:Watermelon:Oman	PQ399662:WmB:2023:Melon:Arizona:USA
43	HG941655:WmA:2013:Squash:Oman	
44	KJ958911:WmA:2013:Cucumber:Jizan:SaudiArabia	
45	KM820288:WmA:2014:Watermelon:Israel	
46	KM820287:WmA:2014:Watermelon:Israel	
47	KM820286:WmA:2014:Watermelon:Israel	
48	KM820285:WmA:2014:Watermelon:Israel	
49	KM820284:WmA:2014:Watermelon:Israel	
50	KM820283:WmA:2014:Watermelon:Israel	
51	KM820282:WmA:2014:Watermelon:Israel	
52	KM820281:WmA:2014:Watermelon:Israel	
53	KM820280:WmA:2014:Watermelon:Israel	
54	KM820279:WmA:2014:Watermelon:Israel	
55	KM820278:WmA:2014:Watermelon:Israel	
56	KM820277:WmA:2014:Watermelon:Israel	
57	KM820276:WmA:2014:Watermelon:Israel	
58	KM820275:WmA:2014:Watermelon:Israel	
59	KM820274:WmA:2014:Watermelon:Israel	
60	KM820273:WmA:2014:Watermelon:Israel	
61	KM820272:WmA:2014:Watermelon:Israel	
62	KM820271:WmA:2014:Watermelon:Israel	
63	KM820270:WmA:2014:Watermelon:Israel	
64	KM820269:WmA:2014:Watermelon:Israel	
65	KM820268:WmA:2014:Watermelon:Israel	
66	KM820267:WmA:2014:Watermelon:Israel	
67	KM820266:WmA:2014:Watermelon:Israel	
68	KM820265:WmA:2014:Watermelon:Israel	
69	KM820264:WmA:2014:Watermelon:Israel	
70	KM820263:WmA:2014:Watermelon:Israel	
71	KM820262:WmA:2014:Watermelon:Israel	
72	KM820261:WmA:2014:Watermelon:Israel	
73	KM820260:WmA:2014:Watermelon:Israel	
74	KM820259:WmA:2014:Watermelon:Israel	
75	KM820258:WmA:2014:Watermelon:Israel	
76	KM820257:WmA:2014:Watermelon:Israel	
77	KM820256:WmA:2014:Watermelon:Israel	
78	KM820255:WmA:2014:Watermelon:Israel	
79	KM820254:WmA:2014:Watermelon:Israel	
80	KM820253:WmA:2014:Watermelon:Israel	
81	KM820252:WmA:2014:Watermelon:Israel	
82	KM820251:WmA:2014:Watermelon:Israel	
83	KM820250:WmA:2014:Watermelon:Israel	
84	KM820249:WmA:2014:Watermelon:Israel	
85	KM820248:WmA:2014:Watermelon:Israel	
86	KM820247:WmA:2014:Watermelon:Israel	
87	KM820246:WmA:2014:Watermelon:Israel	
88	KM820245:WmA:2014:Watermelon:Israel	
89	KM820244:WmA:2014:Watermelon:Israel	
90	KM820243:WmA:2014:Watermelon:Israel	

91	KM820242:WmA:2014:Watermelon:Israel	
92	KM820241:WmA:2014:Watermelon:Israel	
93	KM820240:WmA:2014:Watermelon:Israel	
94	KM820239:WmA:2014:Watermelon:Israel	
95	KM820238:WmA:2014:Watermelon:Jordan	
96	KM820237:WmA:2014:Watermelon:Jordan	
97	KM820236:WmA:2014:Watermelon:Jordan	
98	KM820235:WmA:2014:Watermelon:Jordan	
99	KM820234:WmA:2014:Watermelon:Jordan	
100	KM820233:WmA:2014:Watermelon:Jordan	
101	KM820232:WmA:2014:Watermelon:Jordan	
102	KM820231:WmA:2014:Watermelon:Jordan	
103	KM820230:WmA:2014:Watermelon:Jordan	
104	KM820229:WmA:2014:Watermelon:Jordan	
105	KM820228:WmA:2014:Watermelon:Jordan	
106	KM820227:WmA:2014:Watermelon:Jordan	
107	KM820226:WmA:2014:Watermelon:Jordan	
108	KM820225:WmA:2014:Watermelon:Jordan	
109	KM820224:WmA:2014:Watermelon:Jordan	
110	KM820223:WmA:2014:Watermelon:Jordan	
111	KM820222:WmA:2014:Watermelon:Jordan	
112	KM820221:WmA:2014:Watermelon:Jordan	
113	KM820220:WmA:2014:Watermelon:Jordan	
114	KM820219:WmA:2014:Watermelon:Jordan	
115	KM820218:WmA:2014:Watermelon:Jordan	
116	KM820217:WmA:2014:Watermelon:Jordan	
117	KM820216:WmA:2014:Watermelon:Jordan	
118	KM820215:WmA:2014:Watermelon:Jordan	
119	KM820214:WmA:2014:Watermelon:Jordan	
120	KM820213:WmA:2014:Watermelon:Jordan	
121	KM820211:WmA:2014:Watermelon:Jordan	
122	KM820210:WmA:2014:Watermelon:Jordan	
123	KM820209:WmA:2014:Watermelon:Jordan	
124	KM820208:WmA:2014:Watermelon:Jordan	
125	KM820207:WmA:2014:Watermelon:Jordan	
126	KM820206:WmA:2014:Watermelon:Jordan	
127	KM820205:WmA:2014:Watermelon:Jordan	
128	KM820204:WmA:2014:Watermelon:Jordan	
129	KM820203:WmA:2014:Watermelon:Jordan	
130	KM820202:WmA:2014:Watermelon:Jordan	
131	KM820201:WmA:2014:Watermelon:Jordan	
132	KM820200:WmA:2014:Watermelon:Jordan	
133	KM820199:WmA:2014:Watermelon:Jordan	
134	KM820198:WmA:2014:Watermelon:Jordan	
135	KM820197:WmA:2014:Watermelon:Jordan	
136	KM820196:WmA:2014:Watermelon:Jordan	
137	KM820195:WmA:2014:Watermelon:Jordan	
138	KM820194:WmA:2014:Watermelon:Jordan	
139	KM820193:WmA:2014:Watermelon:Jordan	
140	KM820192:WmA:2014:Watermelon:Jordan	
141	KM820191:WmA:2014:Watermelon:Jordan	
142	KM820190:WmA:2014:Watermelon:Jordan	
143	KM820189:WmA:2014:Watermelon:Jordan	
144	KM820188:WmA:2014:Watermelon:Jordan	
145	KM820187:WmA:2014:Watermelon:Jordan	
146	KM820186:WmA:2014:Watermelon:Jordan	
147	KM820185:WmA:2014:Watermelon:Jordan	

148	KM820184:WmA:2014:Watermelon:Jordan	
149	KM820212:WmA:2014:Watermelon:Jordan	
150	KJ958912:WmA:2012:Zucchini:AlAhsa:SaudiArabia	
151	PP622785:WmA:2021:Melon:Mexico	
152	KT272773:WmA:2010:Cucurbit:Iran	

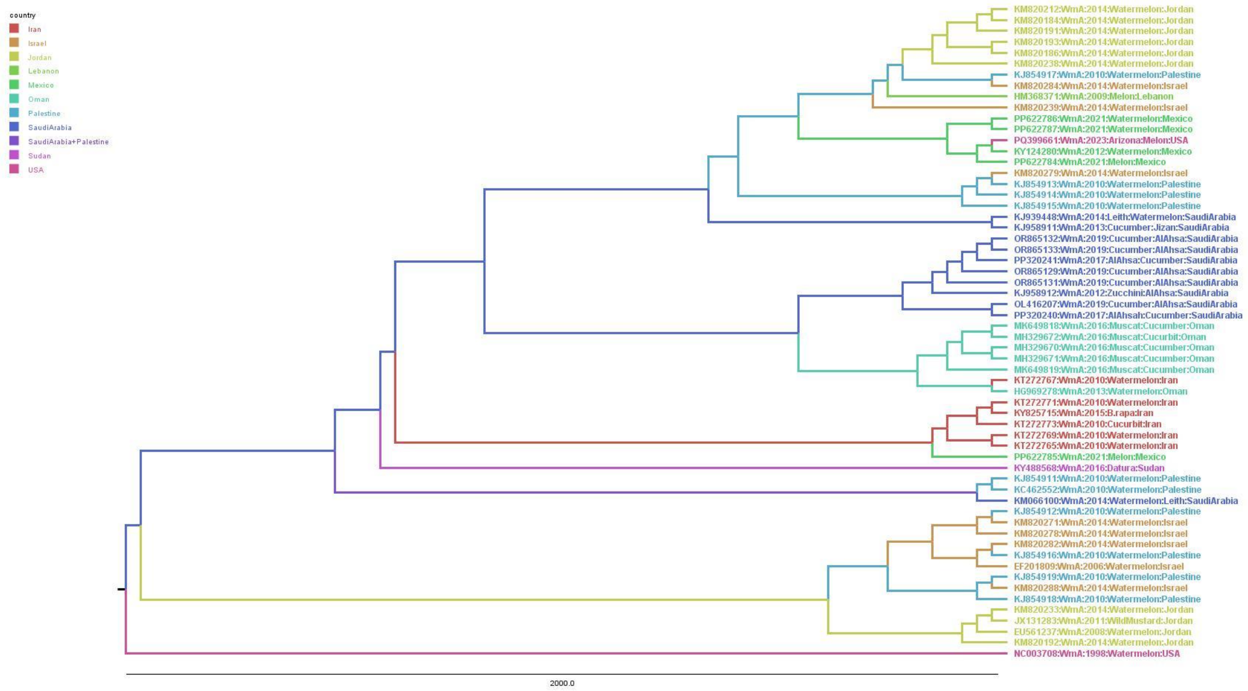

Figure S1. Maximum clade credibility (MCC) tree of WmA (n=60) isolates based on full genome sequences. The time-scaled phylogenetic tree was inferred using BEAST, employing a discrete phylogeographic model to reconstruct viral relationships and spatiotemporal spread. Branches and tip labels are color-coded according to the country of origin of each isolate, as indicated in the legend (left). The x-axis represents time in years (substitution rate scale). The tree highlights well-supported geographic structuring with clear clustering of isolates from Saudi Arabia, Jordan, Oman, and Palestine, indicating local diversification and limited cross-border transmission events.

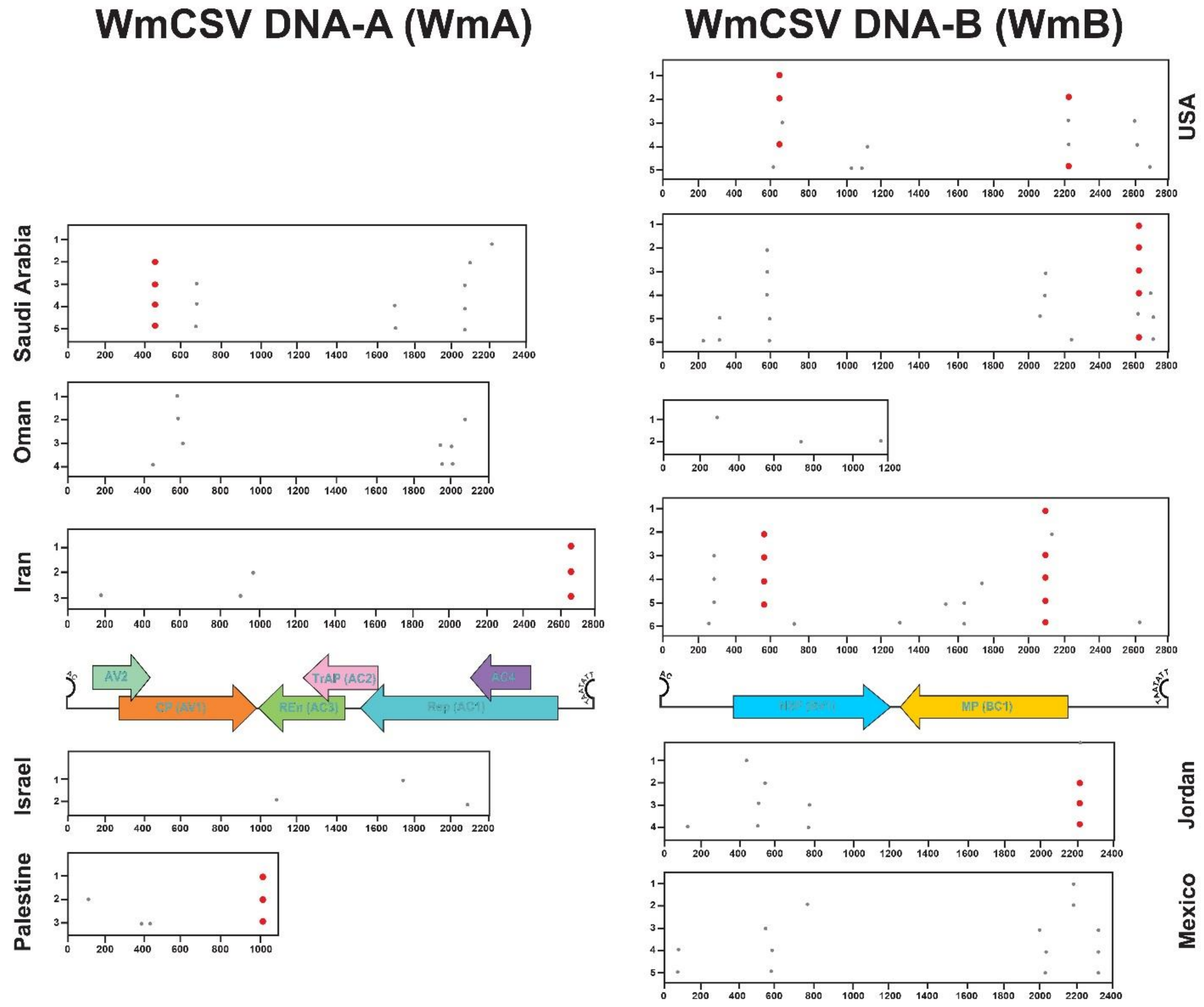

Figure S2. Distribution of recombination breakpoints in datasets of WmA and WmB in different countries. High-confidence breakpoints (supported by high AICc scores) are marked by large red dots, while less reliable breakpoints (lower AICc scores) appear as small gray dots. The x-axis displays nucleotide positions, and the y-axis shows the breakpoint count. The linear genome structures of WmA and WmB are illustrated for reference.

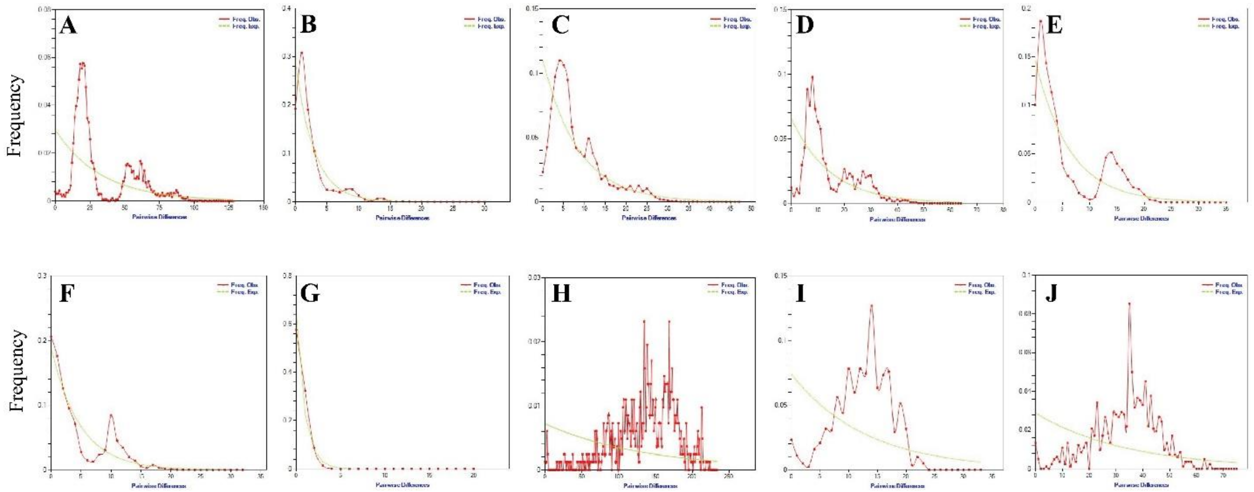

Figure S3. Mismatch distribution curves of WmA (A), AV2 (B), CP (C), Rep (D), AC2 (E), AC3 (F), AC4 (G), WmB (H), NSP (I), and MP (J). The dash line shows the empirical pairwise-difference distribution whereas the solid line is an equilibrium distribution with the same mean value.
